# Characterization of the complete chloroplast genome sequence of medicinal plant: *Potentilla bifurca* (Rosaceae)

**DOI:** 10.1080/23802359.2020.1852901

**Published:** 2021-01-16

**Authors:** Yi Liu, Shengxia Xiang, Xi Fu

**Affiliations:** a Hospital of Chengdu, University of Traditional Chinese Medicine, Chengdu, Sichuan, P. R. China; b Department of Oncology, Sichuan Integrative Medicine Hospital, Chengdu, Sichuan, P. R. China

**Keywords:** Chloroplast genome, phylogenetic analysis, Rosaceae, *Potentilla bifurca*

## Abstract

*Potentilla bifurca* is a medicinal plant, the root extracts have been applied for the treatment of certain viral infections as folk medicinal herbs. The complete chloroplast genome sequence of is 155,841 bp in length, contains 128 complete genes, including 84 protein-coding genes (84 PCGs), 8 ribosomal RNA genes (4 rRNAs), and 37 tRNA genes (37 tRNAs). The overall GC content of cp DNA is 37.1%, the corresponding values of the LSC, SSC, and IR regions are 35.0%, 31.0%, and 42.8%. Phylogenetic tree shows that *P. bifurca was* identified as the basal clade of Potentilla.

*Potentilla bifurca* Linnaeus belongs to genus *Potentilla* (Rosaceae), which is distributed in temperate and alpine zones of the Northern hemisphere. *Potentilla bifurca* has been known for its decorative value and curative properties (Eriksson et al. 1998). Extracts from the aerial and underground parts have been widely applied in Chinese traditional medicine. It is applied externally to treat antioxidant, antitumor and antiulcerogenic diseases (Gürbüz et al. 2005; Tomczyk et al. 2010; Tomczyk et al. 2013). In Tibet, *P. bifurca* root extracts are also used for the treatment of certain viral infections as folk medicinal herbs (Zhao et al. 2008). However, the chloroplast genome of *P. bifurca* has not been reported despite its important medical value. In this study, we assembled the complete chloroplast genome of *P. bifurca*, hoping to lay a foundation for further research.

Fresh leaves of *P. bifurca* were collected from Xiji (Guyuan, Ningxia, China; coordinates: 105°54′E, 35°50′N) and dried with silica gel. The voucher specimen was stored in Sichuan University Herbarium with the number is QTPLJQ14383036. Total genomic DNA was extracted with a modified CTAB method (Doyle and Doyle 1987) and a 350-bp library was constructed. This library was sequenced on the Illumina NovaSeq 6000 system with 150 bp paired-end reads. We obtained 10 million high quality pair-end reads for *P. bifurca*, and after removing the adapters, the remained reads were used to assemble the complete chloroplast genome by NOVOPlasty (Dierckxsens et al. 2017). The complete chloroplasts genome sequence of *D. longicalyx* was used as a reference. Plann v1.1 (Huang and CronK 2015) and Geneious v11.0.3 (Kearse et al. 2012) were used to annotate the chloroplasts genome and correct the annotation.

The total plastome length of *P. bifurca* (MT712015) is 155,841 bp, exhibits a typical quadripartite structural organization, consisting of a large single copy (LSC) region of 85,128 bp, two inverted repeat (IR) regions of 26,061 bp and a small single copy (SSC) region of 18,591 bp. The cp genome contains 129 complete genes, including 84 protein-coding genes, 8 ribosomal RNA genes, and 37 tRNA genes. Most genes occur in a single copy, while 16 genes occur in double, including all rRNAs (4.5S, 5S, 16S, and 23S rRNA), 7 tRNAs (*trnA-UGC*, *trnI-CAU*, *trnI-GAU*, *trnL-CAA*, *trnN-GUU*, *trnR-ACG*, and *trnV-GAC*), and 5 PCGs (*rps7*, *ndhB*, *ycf2*, *rpl2* and *rpl23*). The overall GC content of cp DNA is 37.1%, the corresponding values of the LSC, SSC, and IR regions are 35.0%, 31.0%, and 42.8%.

In order to further clarify the phylogenetic position of *P. bifurca*, plastome of 8 representative *Potentilla* species were obtained from NCBI to reconstruct the plastome phylogeny, with *Fragaria viridis* as an outgroup. All the sequences were aligned using MAFFT v.7.313 (Katoh and Standley 2013) and maximum likelihood phylogenetic analyses were conducted using RAxML v.8.2.11 (Stamatakis 2014) under GTRCAT model with 500 bootstrap replicates. The phylogenetic tree shows that *P. bifurca* was the basal clade of *Potentilla* (Figure 1).

**Figure 1.**
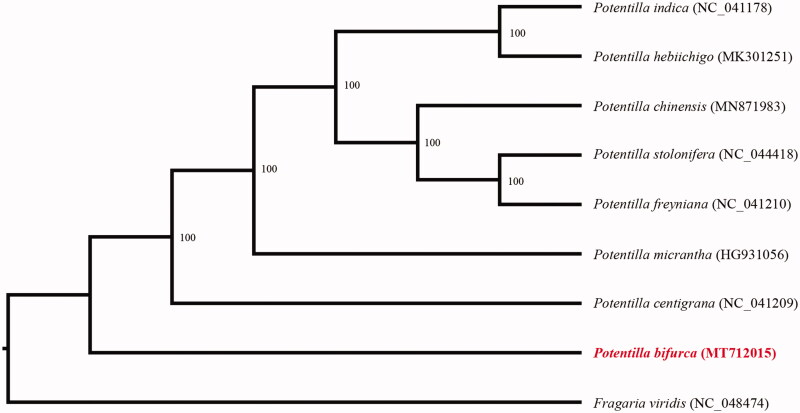
Phylogenetic relationships of *Potentilla* species using whole chloroplast genome. GenBank accession numbers: *Potentilla bifurca* (MT712015), *Potentilla centigrana* (NC_041209), *Potentilla chinensis* (MN871983), *Potentilla freyniana* (NC_041210), *Potentilla hebiichigo* (MK301251), *Potentilla indica* (NC_041178), *Potentilla micrantha* (HG931056), *Potentilla stolonifera* (NC_044418), *Fragaria viridis* (NC_048474).

## Data Availability

The raw sequencing data were deposited in the NCBI Sequence Read Archive (SRA) database, the accession number is SAMN16562210. The final genome assembly are openly available in GenBank of NCBI at https://www.ncbi.nlm.nih.gov, the reference number is MT712015.
